# Supplementary material for: Senescent preosteoclast secretome promotes metabolic syndrome associated osteoarthritis through cyclooxygenase 2
Source: eLife. 2022 Jul 26;11:e79773. doi: 10.7554/eLife.79773 (PMC9365389; doi:10.7554/eLife.79773)
Supplement: Supplementary file 1. — (A) Baseline characteristics of the participants according to presence of metabolic syndrome-associated OA (MetS+ PTOA– versus PTOA+ MetS–) before and after propensity score matching.(B) Baseline characteristics of human COX2 inhibitor and non-selective NSAID users included in the study, before and after propensity score matching. Matched participants were includde in the analysis of COX2 inhibitor use association with OA outcomes, according to its phenotype.(C) Osteoarthritis Initiative (OAI) datasets used in the study. (D) Flowchart outlining the selection criteria and PS-matching process according to the presence of metabolic syndrome-associated OA (MetS-OA) and post-traumatic OA (PTOA) in Osteoarthritis initiative participants. (E) Flowchart outlining the selection criteria and PS-matching process of human COX2 inhibitor and non-selective NSAID users from the Osteoarthritis initiative dataset. [file elife-79773-supp1.docx]

**SUPPLEMENTARY MATERIAL**

**Supplementary File 1A** Baseline characteristics of the participants according to presence of metabolic syndrome-associated OA (MetS^+^ PTOA^–^ versus PTOA^+^ MetS^–^) before and after propensity score matching.

|  | **Eligible OAI participants before matching** | |  | **PS-matched participants** | |  |
| --- | --- | --- | --- | --- | --- | --- |
|  | **PTOA** | **MetS-OA** |  | **PTOA** | **MetS-OA** |  |
|  | **N: 412** | **N: 218** | **SMD** | **N: 169** | **N: 169** | **SMD** |
| **Variables included in the PS matching model** | | | | | | |
| **Age (year), mean (SD)** | 58.86 (8.49) | 65.28 (8.18) | **0.769** | 63.36 (8.31) | 63.75 (7.97) | 0.049 |
| **Sex, Female, N (%)** | 229 (55.6) | 127 (58.3) | 0.054 | 95 (56.2) | 101 (59.8) | 0.072 |
| **Non-white race, N (%) ^†^** | 54 (13.1) | 44 (20.2) | **0.191** | 30 (17.8) | 37 (21.9) | 0.098 |
| **BMI (kg/m^2^), mean (SD)** | 28.99 (4.77) | 30.59 (4.26) | **0.353** | 30.30 (4.43) | 30.26 (4.28) | 0.009 |
| **Smoking, current smoker, N (%)** | 18 (4.4) | 10 (4.6) | 0.011 | 10 (5.9) | 9 (5.3) | 0.026 |
| **Alcohol use, ≥1/week N (%)** | 200 (48.5) | 77 (35.3) | **0.270** | 63 (37.3) | 67 (39.6) | 0.049 |
| **PASE score, mean (SD)** | 178.4 (89.55) | 148.9 (74.4) | **0.358** | 153.19 (78.3) | 154.56 (73.4) | 0.018 |
| **KL grade, N (%)** |  |  | **0.291** |  |  | **0.159** |
| Grade 0 | 63 (15.3) | 36 (16.5) |  | 28 (16.6) | 22 (13.0) |  |
| Grade 1 | 108 (26.2) | 75 (34.4) |  | 49 (29.0) | 57 (33.7) |  |
| Grade 2 | 103 (25.0) | 52 (23.9) |  | 47 (27.8) | 47 (27.8) |  |
| Grade 3 | 94 (22.8) | 46 (21.1) |  | 32 (18.9) | 34 (20.1) |  |
| Grade 4 | 44 (10.7) | 9 (4.1) |  | 13 (7.7) | 9 (5.3) |  |
| **Medial JSN score, N (%)** |  |  | **0.225** |  |  | **0.132** |
| Grade 0 | 193 (46.8) | 100 (45.9) |  | 77 (45.6) | 77 (45.6) |  |
| Grade 1 | 99 (24.0) | 66 (30.3) |  | 50 (29.6) | 51 (30.2) |  |
| Grade 2 | 86 (20.9) | 44 (20.2) |  | 29 (17.2) | 33 (19.5) |  |
| Grade 3 | 34 (8.3) | 8 (3.7) |  | 13 (7.7) | 8 (4.7) |  |
| **Variables not included in the PS matching model** | | | | | | |
| **History of knee injury, N (%)** | 412 (100.0) | 0 (0.0) | – | 169 (100.0) | 0 (0.0) | – |
| **WOMAC pain score, mean (SD)** | 14.42 (15.19) | 14.42 (15.72) | 0.001 | 14.85 (15.36) | 15.88 (16.23) | 0.065 |
| **Cardio/Cerebrovascular diseases, N (%)** | 21 (5.2) | 19 (9.0) | **0.148** | 12 (7.3) | 14 (8.5) | 0.045 |
| **Hypertension, N (%)‡** | 143 (34.7) | 208 (95.4) | **1.651** | 72 (42.6) | 162 (95.9) | **1.413** |
| **Diabetes Mellitus, N (%)‡** | 3 (0.7) | 84 (39.1) | **1.094** | 1 (0.6) | 62 (37.3) | **1.061** |
| **Dyslipidemia, N (%)‡** | 57 (13.8) | 185 (84.9) | **2.018** | 23 (13.6) | 142 (84.0) | **1.984** |
| **Abdominal obesity N (%)‡** | 360 (87.6) | 218 (100.0) | **0.532** | 153 (91.1) | 169 (100.0) | **0.443** |

BMI: body mass index, JSN: joint space narrowing, KL: Kellgren-Lawrence, MetS: metabolic syndrome, OAI: Osteoarthritis Initiative, PASE: physical activity scale for the elderly, PS: propensity score; SD: Standard deviation, SMD: standardized mean difference, WOMAC: Western Ontario and McMaster Universities Arthritis Index. Quantitative variables are shown in mean (± SD), and qualitative variables are shown in number (%). Participants with MetS and no history of knee trauma were considered as having MetS-OA, and participants with a history of knee trauma but not MetS were regarded as having PTOA. A significant difference for SMD was defined as ≥0.1 and shown as bold.

**†**Race of participants was categorized as white and non–white considering the small number of participants in each non-white race group.

‡Components of MetS are defined by the International Diabetes Federation as follows: 1) hypertension: ≥130 mm Hg systolic blood pressure or ≥85 mm Hg at baseline physical examination diastolic blood pressure or taking blood pressure-lowering medication indicated in the medication history; 2) diabetes: self-reported diabetes or use of oral or injective anti-diabetic medications indicated in the participant’s medication history; 3) dyslipidemia: use of lipid-lowering medications indicated in the participant’s medication history; and 4) abdominal obesity: waist circumference of ≥94 cm in men and ≥80 cm in women. According to the International Diabetes Federation criteria, MetS is defined as abdominal obesity and at least 2 of the 3 other criteria (hypertension, dyslipidemia, and diabetes).

**Supplementary File 1B** Baseline characteristics of human COX2 inhibitor and non-selective NSAID users included in the study, before and after propensity score matching. Matched participants were includde in the analysis of COX2 inhibitor use association with OA outcomes, according to its phenotype.

|  | **Eligible OAI participants before matching** | | | |  | | **PS-matched participants** | | | | | |
| --- | --- | --- | --- | --- | --- | --- | --- | --- | --- | --- | --- | --- |
|  | **Non-selective NSAID users** | | **COX2 Inhibitor users** | | **SMD** | | **Non-selective NSAID users** | | **COX2 Inhibitor users** | | **SMD** | |
|  | N: 488 | | N: 315 | |  | | N: 239 | | N: 239 | |  | |
| **Variables included in the PS-matching** | |  | |  | |  | |  | |  | |  |
| **Age, mean (SD)** | 60.23 (8.98) | | 61.55 (9.01) | | **0.147** | | 60.87 (9.16) | | 61.29 (8.80) | | 0.047 | |
| **Sex, Female, N (%)** | 330 (67.6) | | 220 (69.8) | | 0.048 | | 173 (72.5) | | 175 (73.2) | | 0.019 | |
| **Non-white race, N (%)^†^** | 147 (30.2) | | 44 (14.0) | | **0.400** | | 36 (15.2) | | 34 (14.2) | | 0.027 | |
| **BMI, mean (SD)** | 29.87 (5.25) | | 28.74 (4.99) | | **0.221** | | 28.98 (5.19) | | 29.31 (5.04) | | 0.064 | |
| **History of knee injury, N (%)** | 147 (30.3) | | 86 (27.4) | | 0.065 | | 66 (27.6) | | 68 (28.6) | | 0.021 | |
| **WOMAC pain score, mean (SD)** | 3.78 (4.25) | | 3.24 (3.61) | | **0.137** | | 3.43 (4.07) | | 3.14 (3.69) | | 0.076 | |
| **Variables not included in the PS-matching** | |  | |  | |  | |  | |  | |  |
| **KL grade, N (%)** |  | |  | | **0.142** | |  | |  | | 0.061 | |
| Grade 0 | 147 (30.1) | | 97 (30.8) | |  | | 72 (30.1) | | 72 (30.1) | |  | |
| Grade 1 | 81 (16.6) | | 62 (19.7) | |  | | 36 (15.1) | | 38 (15.9) | |  | |
| Grade 2 | 160 (32.8) | | 87 (27.6) | |  | | 80 (33.5) | | 74 (31.0) | |  | |
| Grade 3 | 79 (16.2) | | 50 (15.9) | |  | | 37 (15.5) | | 40 (16.7) | |  | |
| Grade 4 | 21 (4.3) | | 19 (6.0) | |  | | 14 (5.9) | | 15 (6.3) | |  | |
| **Medial JSN score, N (%)** |  | |  | | 0.070 | |  | |  | | 0.073 | |
| Grade 0 | 272 (60.4) | | 175 (60.6) | |  | | 138 (61.1) | | 134 (61.8) | |  | |
| Grade 1 | 109 (24.2) | | 66 (22.8) | |  | | 55 (24.3) | | 48 (22.1) | |  | |
| Grade 2 | 57 (12.7) | | 37 (12.8) | |  | | 25 (11.1) | | 28 (12.9) | |  | |
| Grade 3 | 12 (2.7) | | 11 (3.8) | |  | | 8 (3.5) | | 7 (3.2) | |  | |
| **PASE, mean (SD)** | 154.21 (84.59) | | 160.99 (82.34) | | 0.081 | | 157.57 (82.54) | | 162.05 (83.21) | | 0.054 | |
| **WOMAC total score, mean (SD)** | 18.74 (19.15) | | 15.93 (16.44) | | **0.158** | | 16.41 (17.88) | | 15.97 (17.27) | | 0.025 | |
| **Abdominal Obesity, N (%)** | 444 (91.4) | | 292 (92.7) | | 0.050 | | 218 (91.6) | | 227 (95.0) | | **0.135** | |
| **Cardio/Cerebrovascular diseases, N (%)** | 16 (3.3) | | 19 (6.1) | | **0.129** | | 6 (2.5) | | 15 (6.3) | | **0.186** | |
| **Diabetes, N (%)** | 44 (9.3) | | 22 (7.1) | | 0.081 | | 17 (7.2) | | 20 (8.5) | | 0.046 | |
| **MetS by IDF criteria, N (%)**^♦^ | 107 (21.9) | | 75 (23.8) | | 0.045 | | 46 (19.2) | | 64 (26.8) | | **0.180** | |
| **COX2 Inhibitor medication name, N (%)** |  | |  | | – | |  | |  | | – | |
| Celecoxib | – | | 149 (47.3) | |  | | – | | 113 (47.3) | |  | |
| Etoricoxib | – | | 2 (0.6) | |  | | – | | 2 (0.8) | |  | |
| Lumiracoxib | – | | 2 (0.6) | |  | | – | | 1 (0.4) | |  | |
| Rofecoxib | – | | 69 (21.9) | |  | | – | | 51 (21.3) | |  | |
| Valdecoxib | – | | 93 (29.5) | |  | | – | | 72 (30.1) | |  | |

BMI: Body mass index, JSN: Joint Space Narrowing, KL: Kellgren-Lawrence, PASE: physical activity scale for the elderly, PS: propensity score, SMD: Standardized mean difference, SD: Standard deviation. Quantitative variables are shown in mean (± standard deviation), and qualitative variables are shown in number (% percent). Data are presented as numbers of knees. A significant difference for SMD was defined as ≥ 0.1.

♦Components of MetS are defined by the International Diabetes Federation as follows: 1) hypertension: ≥130 mm Hg systolic blood pressure or ≥85 mm Hg at baseline physical examination diastolic blood pressure or taking blood pressure-lowering medication indicated in the medication history; 2) diabetes: self–reported diabetes or use of oral or injective anti-diabetic medications indicated in the participant’s medication history; 3) dyslipidemia: use of lipid-lowering medications indicated in the participant’s medication history; and 4) abdominal obesity: waist circumference of ≥94 cm in men and ≥80 cm in women. According to the International Diabetes Federation criteria, MetS is defined as abdominal obesity and at least 2 of the 3 other criteria (hypertension, dyslipidemia, and diabetes). In further analysis patients were stratified for presence of MetS-OA (Table 2 and 3).

**†**Race of participants was categorized as white and non-white considering the small number of participants in each non-white race group.

| **Supplementary File 1C.** Osteoarthritis Initiative (OAI) datasets used in the study | | |
| --- | --- | --- |
| **Dataset** | **Visit** | **Release version** |
| All clinical | allclinical00 | 0.2.2 |
| (Data of all clinical information and NASS incidence) | allclinical01 | 1.2.1 |
|  | allclinical03 | 3.2.1 |
|  | allclinical05 | 5.2.1 |
|  | allclinical06 | 6.2.1 |
|  | allclinical07 | 7.2.1 |
|  | allclinical08 | 8.2.2 |
|  | allclinical09 | 9.2.1 |
|  | allclinical10 | 10.2.2 |
| Medical inventory form (MIF) | MIF00 | 0.2.2 |
| (Data of medication use) | MIF01 | 1.2.1 |
|  | MIF03 | 3.2.1 |
|  | MIF05 | 5.2.1 |
|  | MIF06 | 6.2.1 |
|  | MIF07 | 7.2.1 |
|  | MIF08 | 8.2.2 |
|  | MIF09 | 9.2.1 |
|  | MIF10 | 10.2.2 |
| Enrollees | Enrollees | 25 |
| (Data regarding baseline enrollment of OAI participants) |  |  |
| Knee X-ray semi-quantitative reading (Kxr sq) | Kxr sq 00 | 0.8 |
| MRI tracking and QA | mri00 | 0.2.2 |
| (Data regarding availability of MRI and | mri03 | 3.2.1 |
| quality assessment) | mri06 | 6.2.1 |
| Foundation for the National Institute of Health | FNIH | – |
| Project no. 30 | Project no. 30 | – |
| Projects no. 63A-63F | Projects no. 63A-63F | – |
| Pivotal OAI MRI Analyses | POMA | – |
| (OAI ancillary studies with MOAKS BML readings) |  |  |
| Outcomes | outcome99 | 10 |
| (Data regarding all knee OA outcomes including incidence and JSN progression) |  |  |
| BML: Bone marrow lesion, JSN: Joint space narrowing, MOAKS: MRI Osteoarthritis Knee Score, NASS: Non-acceptable symptomatic state, OAI: Osteoarthritis initiative, QA: Quality assessment | | |

**Supplementary File 1D** Flowchart outlining the selection criteria and PS-matching process according to the presence of metabolic syndrome-associated OA (MetS-OA) and post-traumatic OA (PTOA) in Osteoarthritis initiative participants.

**
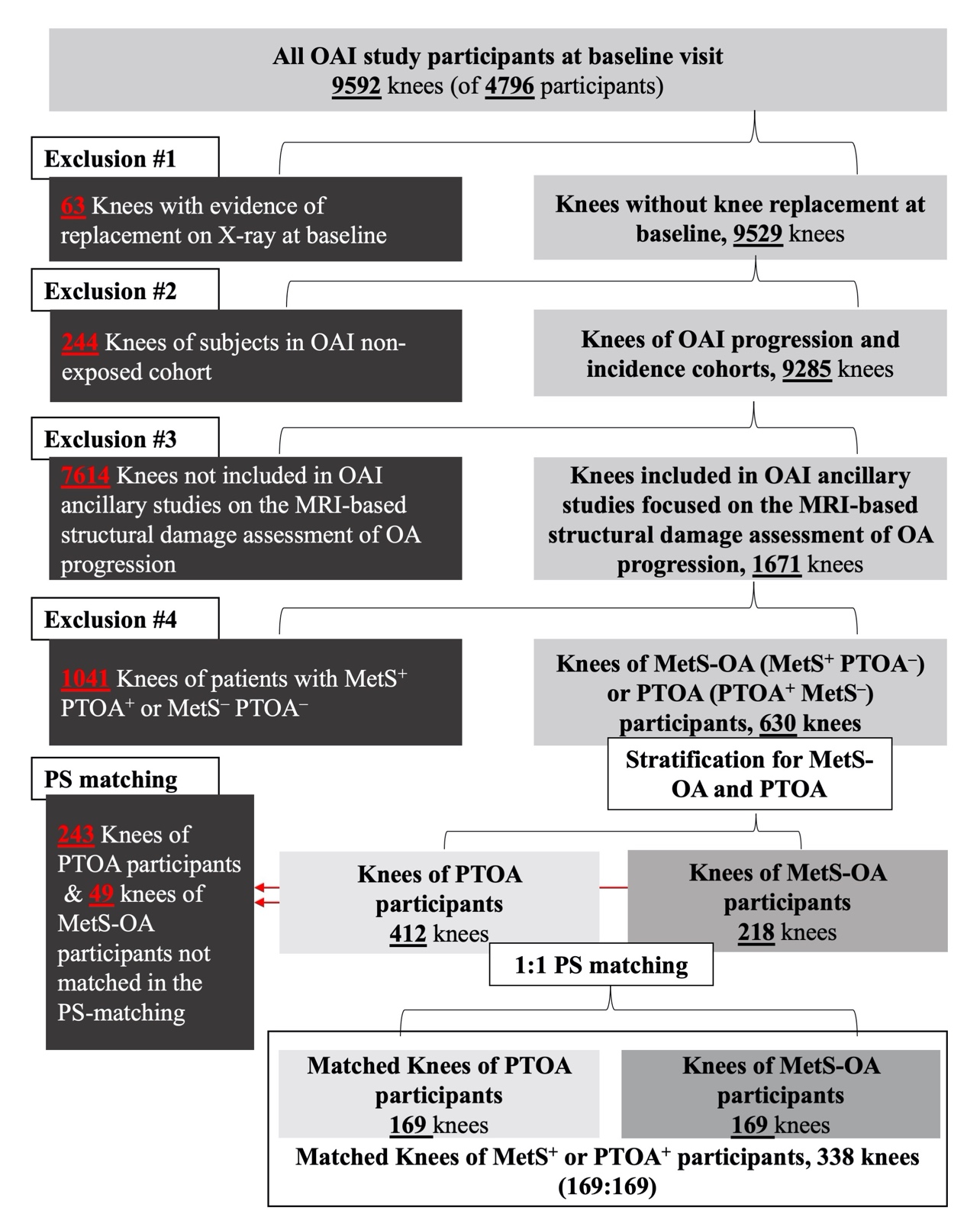
**

KL: Kellgren-Lawrence, MetS: Metabolic syndrome, OAI: Osteoarthritis Initiative, PS: propensity score, PTOA: Post-traumatic osteoarthritis**Supplementary File 1E** Flowchart outlining the selection criteria and PS-matching process of human COX2 inhibitor and non-selective NSAID users from the Osteoarthritis initiative dataset.


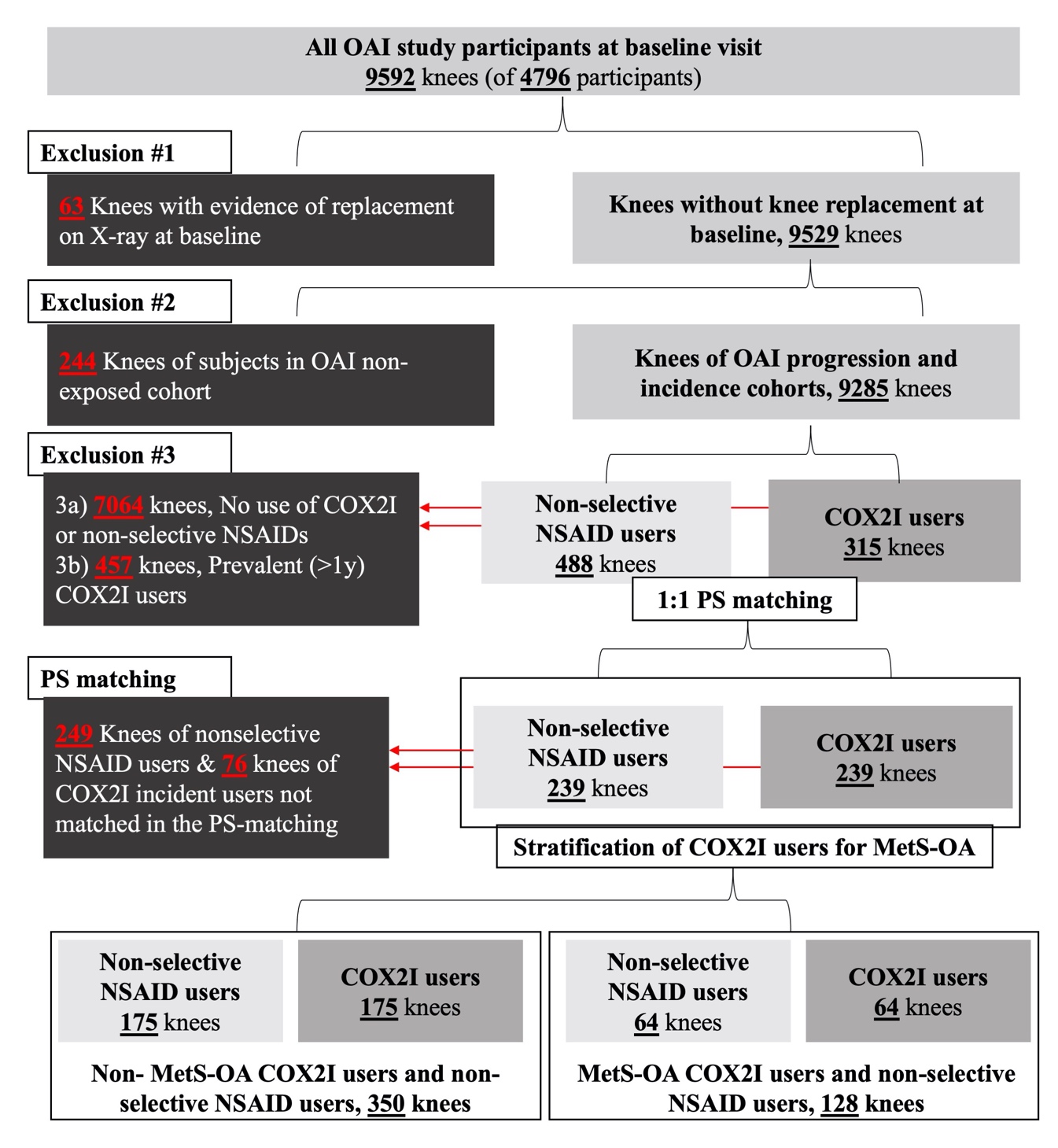


COX2I: Cyclooxygenase 2 inhibitor, KL: Kellgren-Lawrence, MetS: Metabolic syndrome, NSAID: Non-Steroidal Anti-Inflammatory Drug, OAI: Osteoarthritis Initiative, PS: propensity score
